# Coronavirus disease 2019: Lessons, risks and challenges

**DOI:** 10.7196/AJTCCM.2020.v26i2.068

**Published:** 2020-04-24

**Authors:** G Richards, S Stacey

**Affiliations:** ^1^ Department of Critical Care, School of Clinical Medicine, Faculty of Health Sciences, University of the Witwatersrand, Johannesburg, South Africa; ^2^ Division of Infectious Diseases, Department of Medicine, School of Clinical Medicine, Faculty of Health Sciences, University of Witwatersrand, Johannesburg, South Africa

**Keywords:** covid-19, coronavirus

## Abstract

There have been several viral pandemics that have swept the globe over the past century. The latest one is the COVID-19 pandemic caused by the severe acute respiratory syndrome coronavirus 2 (SARS-CoV-2). In this mini review, we outline the epidemiology, clinical presentation, management and prognosis of COVID-19. The pandemic is part of a rapidly changing landscape and it remains to be seen how events will unfold in South Africa, where there is a large reservoir of young people with sub-optimal lung immunity due to several causes, including HIV, post-tuberculous lung disease, smoking, biomass fuel exposure and poor socioeconomic circumstances.

*Afr J Thoracic Crit Care Med* 2020;26(2):Published online 24 April 2020. https://doi.org/10.7196/AJTCCM.2020.v26i2.068

On 30 January 2020, the World Health Organization (WHO) declared the disease (COVID-19) caused by the novel severe acute respiratory syndrome coronavirus 2 (SARS-CoV-2) a global public health emergency of international concern, after 98 cases had been confirmed in countries outside of China, and 4 cases of human-to-human transmission had been confirmed.[1] A week later, the number of cases outside China had risen to 270 in 24 countries, with 1 death confirmed in the Philippines. The apparent reason for the declaration of an emergency was not the gravity of the situation in China, which was expected to contain the outbreak, but rather the possibility of spread to countries with weaker healthcare systems such as those in Africa, many of which do not have the capacity to cope effectively with epidemic transmission on the continent. Since those early days of the epidemic, there has been unprecedented and rapid spread within China and to many countries without. This changes minute-to-minute, and for an updated record of cases see https://www.worldometers.info/coronavirus/. There have been several recent reports and reviews on COVID-19; a recent report by Stacey and Richards[2] has been updated and is published here.

SARS-CoV-2 is a pathogen that is phylogenetically similar to two previous zoonotic coronaviruses, severe acute respiratory syndrome coronavirus 1 (SARS-CoV-1) and the Middle East respiratory syndrome coronavirus (MERSCoV), which caused epidemics in 2002 and 2012, respectively.[3] However, according to Anthony Fauci, Director of the National Institute of Allergy and Infectious Diseases at the National Institutes of Health (NIH) in the USA, in a recent interview with Howard Bauchner, editor-in-chief of the *JAMA*, this virus is behaving epidemiologically in a manner more reminiscent of a severe influenza season rather than either of the other CoV outbreaks this century.[4]

At the time of writing, the number of new cases in China and South Korea is declining, and it is expected that it will be controlled there as isolation and control is easier in countries where the population is intrinsically obedient and the State relatively authoritarian. Of more concern is the expanding situation in Italy where, despite a first-world healthcare system, restriction of movement and mandatory isolation has been more difficult to achieve. This has been particularly evident in the spread to other European countries from Italy, such as Germany, France, Spain and the UK, where numbers and mortality, particularly in the latter three, have expanded exponentially.

Researchers have identified Egypt, Algeria and South Africa (SA) as the African countries most at risk for importation of cases.[5] It is estimated that China's financial involvement with Africa has grown substantially since the turn of the century, with trade increasing from a very small amount in 2000 to USD170 billion in 2013, surpassing the USA as sub-Saharan Africa's largest trading partner in 2009.[6] Given the considerable trade and tourist ties that exist between SA and China, it is reasonable to expect cases of coronavirus infection to occur in SA. In addition, the National Institute for Communicable Diseases (NICD) has broadened the case definition to include close contact with any person with a history of travel to areas with 'presumed ongoing community transmission' of the virus.[7] At present, the majority of these will have travelled from Italy and Europe and it will be important to keep a close eye on the evolving epidemiology of the disease in the weeks to come.

While case fatality rates (CFRs) in the SARS-CoV-1 and MERS-CoV outbreaks were 7 - 10% and 30%, respectively,[8] in the current epidemic, the CFR is estimated to be ~1 - 2% of confirmed cases, although this may not take into account a larger denominator that includes asymptomatic or mildly symptomatic cases not presenting to healthcare facilities for confirmation of the diagnosis. However, the reproduction number ($R_0$) for COVID-19 is currently thought to be 2.68, which is higher than that of SARS-CoV-1, MERS-CoV and seasonal influenza, and suggests that while this number remains >1 the epidemic will accelerate and would have to be reduced to <1 by appropriate infection control measures, including quarantine and or vaccine in order to halt the pandemic.[9,10] There are anecdotal reports of transmission from asymptomatic patients, but these have yet to be stringently interrogated. The main method of transmission is via droplet spread, and if asymptomatic, the patient is unlikely to be coughing or sneezing.

The clinical features have now been well described and a clearer picture of the spectrum of disease is emerging. In general, symptoms have been typical of CoV respiratory tract infections, and include fever, fatigue, cough and shortness of breath, and range from none to mild or moderate to very severe and life-threatening.[11,12] The incubation period is mostly in the region of 5 - 6 days, but could be as short as 2, and it is recommended that anyone with a significant travel or contact history be monitored for 14 days.[9] In 138 patients hospitalised with COVID-19, 26% were admitted to the intensive care unit (ICU).[13] These patients were more likely to be older and have comorbid conditions, and progression from first symptoms to acute respiratory distress syndrome (ARDS) was on average 8 days. As such, clinicians should be aware that patients presenting early with minimal symptoms should be appropriately monitored for subsequent deterioration, whether at home or in hospital.

A larger retrospective case series of 1 099 patients again demonstrated that the most common presenting symptoms are fever (87.9%) and cough (67%). Disease was more evenly distributed between male and female patients (58.2% and 41.8%, respectively), which differed from previous reports. Non-severe cases outweighed severe cases by a magnitude of 5, with older age and any comorbidity being associated with more severe disease. Overall, severe pneumonia occurred in 15.7% of cases, but the requirement for ICU admission and invasive ventilation was lower than reported elsewhere. Mortality in this study was 1.36%, also lower than current estimates.[14]

In a study of 44 672 confirmed patients from the Chinese Centers for Disease Control and Prevention, the age distribution was as follows: >80 years 3% (1 408 cases); 30 - 79 years 87% (38 680 cases); 20 - 29 years 8% (3 619 cases); 10 - 19 years 1% (549 cases); and <10 years 1% (416 cases).[15] The majority of patients (81%) had mild disease, 14% were severe and 5% were critical. The CFR was 2.3%. This varied according to age, from 14.8% in patients >80 years old to 8.0% in those 70 - 79 years old, and 49.0% in the critically ill. The CFR was also impacted adversely by comorbid illness: 10.5% for cardiovascular disease; 7.3% for diabetes; 6.3% for chronic respiratory disease; 6.0% for hypertension; and 5.6% for cancer. In this study, 3.8% of patients were healthcare personnel, and 14.8% were classified as severe or critical, with five deaths.[15]

All three coronaviruses have the potential to induce excessive, deregulated host immune responses via pathogen-associated molecular patterns. This can result in severe lung injury and ARDS, with ground-glass infiltrates on imaging.[16] This is associated with a ‘cytokine storm’ in the most ill patients, with consequent multiple organ dysfunction.[17] Survivors may have significant pulmonary fibrosis with associated disability and reduced quality of life.

Supportive care appropriate to the clinical severity is advised for the management of COVID-19 infections.[14] Various drug therapies have been postulated to be effective, and recruitment for a study of the protease inhibitor combination lopinavir-ritonavir has already been completed, and a study of the antiviral remdesivir, recently investigated for the treatment of Ebola virus infection, is currently underway and early reports indicate that there are possible benefits.[18] Chloroquine and hydroxychloroquine have good nonspecific antiviral activity *in vitro*, and controlled trials have been initiated using this compounds but initial reports differ regarding whether or not they have any therapeutic benefits.[19,20] Management is otherwise centred around appropriate infection prevention and control, and guidelines issued by the WHO and the Centers for Disease Control and Prevention are excellent in this regard.[21,22]

While the COVID-19 pandemic is likely to be uppermost in the mind of any clinician now faced with an acute respiratory illness of unknown aetiology, it is wise not to forget more common causes of the syndrome, especially as the SA influenza season approaches. As the northern hemisphere influenza season winds down, the USA Centers for Disease Control and Prevention estimate that there have been 22 million cases of influenza in the USA in 2019, 210 000 hospitalisations and 12 000 deaths from a disease which is largely vaccine-preventable.[23] Although the CFR in seasonal influenza is considerably lower than the current estimates for COVID-19, the absolute number of influenza-associated deaths may actually be greater. Healthcare practitioners are advised to receive influenza vaccinations as soon as available, and are expected to provide advice on vaccination to patients, especially those most at risk of severe disease, including pregnant women, HIV-infected individuals, those with comorbid disease, older individuals and young children.

SA, like many other countries, has instituted fever screening at international airports, although many of those with COVID-19 may not have a fever (at least in the early stages). Restrictions have now been imposed on travel to or from areas where there is community spread. Given the magnitude of global traffic, travel restrictions to and from specific countries in the context of pandemic infections were unlikely to have been effective in preventing entry of all infected individuals but could have delayed or minimised additional outbreaks. while new diagnoses in the various epicentres of the pandemic stabilise or start to decline and vaccines or effective antivirals are developed.

It is imperative that we develop a rational approach to suspected cases and infection control in line with local and international guidelines, which will forestall panic among healthcare professionals and the public, and help to avoid the extensive outbreaks in developing countries that the WHO fears.

**Declaration.** None.
**Acknowledgements.** None.
**Author contributions.** Equal contributions.
**Funding.** None.
**Conflicts of interest.** None.

1. World Health Organization. WHO Director-General’s statement on IHR Emergency Committee on Novel Coronavirus (2019-nCoV). https://www.who.int/dg/speeches/detail/who-director-general-s-statement-on-ihr-emergency-committee-on-novel-coronavirus-(2019-ncov) (accessed 8 February 2020).
2. Stacey S, Richards GA. Coronavirus epidemic: A South African perspective. Wits J Clin Med 2020,2(1):1-4. http://doi.org/10.18772/26180197.2020.v2n1a0
3. Paules CI, Marston HD, Fauci AS. Coronavirus infections – more than just the common cold. JAMA 2020(1):23. https://doi:10.1001/jama.2020.0757

4. JN Learning. Global health. The 2019 novel coronavirus outbreak – update from NIAID's Anthony Fauci, MD. https://edhub.ama-assn.org/jn-learning/audioplayer/18217492 (accessed 8 February 2020).
5. Gilbert M, Pullano G, Pinotti G, et al. Preparedness and vulnerability of African countries against importations of COVID-19: A modelling study. Lancet 2020;395(10227):871-877. https://doi.org/10.1016/S0140-6736(20)30411-6
6. The World Bank. Global Economic Prospects June 2015. Linkages between China and sub-Saharan Africa. https://www.worldbank.org/content/dam/Worldbank/GEP/GEP2015b/Global-Economic-Prospects-June-2015-China-and-Sub-Saharan-Africa.pdf (accessed 8 February 2020).
7. National Institute for Communicable Diseases. Novel Coronavirus Infection. http://www.nicd.ac.za/diseases-a-z-index/novel-coronavirus-infection/ (accessed 8 February 2020).
8. Kwok KO, Tang A, Wei VWI, et al. Epidemic models of contact tracing: Systematic review of transmission studies of Severe Acute Respiratory Syndrome and Middle East Respiratory Syndrome. Comput Struct Biotechnol J 2019;17:186-194.
9. Del Rio C, Malani PN. 2019 novel coronavirus – important information for clinicians. JAMA 2020;323(11):1039-1040. https://doi.org/10.1001/jama.2020.1490
10. Coburn BJ, Wagner BG, Blower S. Modeling influenza epidemics and pandemics: Insights into the future of swine flu (H1N1). BMC Med 2009;7:30. https://doi.org/10.1186/1741-7015-7-30
11. Huang C, Wang Y, Li X, et al. Clinical features of patients infected with 2019 novel coronavirus in Wuhan, China. Lancet 2020;395(10223):497-506. https://doi.org/10.1016/S0140-6736(20)301835
12. Chen N, Zhou M, Dong X, et al. Epidemiological and clinical characteristics of 99 cases of 2019 novel coronavirus pneumonia in Wuhan, China: A descriptive study. Lancet 2020;395(10223):507-513. https://doi.org/10.1016/S0140-6736(20)30211-7
13. Wang D, Hu B, Zhu F, et al. Clinical characteristics of 138 hospitalized patients with 2019 novel coronavirus-infected pneumonia in Wuhan, China. JAMA 2020;323(11):1061-1069. https://doi.org/10.1001/jama.2020.1585
14. Guan W, Ni Z, Hu Y, et al. On behalf of China Medical Treatment Expert Group for 1029-nCoV. Clinical characteristics of 2019 novel coronavirus infection in China. https://doi.org/10.1056/NEJMoa2002032
15. Wu Z, McGoogan JM. Characteristics of and important lessons from the coronavirus disease 2019 (COVID-19) outbreak in China. Summary of a report of 72 314 cases from the Chinese Center for Disease Control and Prevention. JAMA 2020;323(13):1239-1242. https://doi.org/10.1001/jama.2020.2648 Accessed 9/3/20
16. Xu Z, Shi L, Wang Y, Zhang J, et al. Pathological findings of COVID-19 associated with acute respiratory distress syndrome. Lancet Respir Med 2020;8(4):420-422. https://doi.org/10.1016/S2213-2600(20)30076-X
17. Yang X, Yu Y, Xu J, et al. Clinical course and outcomes of critically ill patients with SARS-CoV-2 pneumonia in Wuhan, China: A single-centred, retrospective, observational study. Lancet Respir Med 2020;(epub ahead of print). https://doi.org/10.1016/ S2213-2600(20)30079-5
18. Lu H. Drug treatment options for the 2019-newcoronavirus (2019-nCoV). Biosci Trends https://doi.org/10.5582/bst.2020.01020
19. Colson P, Rolain J-M, Lagier J-C, Brouqui P, Raoult D. Chloroquine and hydroxychloroquine as available weapons to fight COVID-19. Int J Antimicrob Agents https://doi.org/10.1016/j.ijantimicag.2020.105932
20. Zumla A, Hui DS, Azhar EI, Memish ZA, Maeure M. Reducing mortality from 2019-nCoV: Host-directed therapies should be an option. Medical Brief 5 February 2020. https://www.medicalbrief.co.za/archives/coronavirus-promising-new-treatments-must-be-explored-immediately/ (accessed 9 March 2020).
21. World Health Organization. Clinical management of severe acute respiratory infection when novel coronavirus (nCoV) infection is suspected. https://www.who.int/publications-detail/clinical-management-of-severe-acute-respiratory-infection-when-novel-coronavirus-(ncov)-infection-is-suspected (accessed 6 March 2020).
22. Centers for Disease Control and Prevention. Coronavirus Disease 2019 (CoVID-19): Interim Clinical Guidance for Management of Patients with Confirmed Coronavirus Disease (COVID-19). https://www.cdc.gov/coronavirus/2019-ncov/hcp/clinical-guidance-management-patients.html (accessed 9 March 2020).
23. Centers for Disease Control and Prevention. Weekly US Influenza Surveillance Report. https://www.cdc.gov/flu/weekly/ (accessed 8 February 2020).

*Accepted 31 March 2020.*
